# From menarche to menopause: A population-based assessment of water, sanitation, and hygiene risk factors for reproductive tract infection symptoms over life stages in rural girls and women in India

**DOI:** 10.1371/journal.pone.0188234

**Published:** 2017-12-05

**Authors:** Kelly K. Baker, Bijaya Padhi, Belen Torondel, Padmalaya Das, Ambarish Dutta, Krushna Chandra Sahoo, Bhabani Das, Robert Dreibelbis, Bethany Caruso, Matthew C. Freeman, Lauren Sager, Pinaki Panigrahi

**Affiliations:** 1 University of Iowa College of Public Health, Iowa City, IA, United States of America; 2 Asian Institute of Public Health, Bhubaneshwar, India; 3 London School of Hygiene and Tropical Medicine, London, United Kingdom; 4 University of Oklahoma, Norman, OK, United States of America; 5 Emory University, Atlanta, GA, United States of America; 6 University of Nebraska Medical Center, Omaha, NE, United States of America; University of North Carolina at Chapel Hill, UNITED STATES

## Abstract

Women face greater challenges than men in accessing water, sanitation, and hygiene (WASH) resources to address their daily needs, and may respond to these challenges by adopting unsafe practices that increase the risk of reproductive tract infections (RTIs). WASH practices may change as women transition through socially-defined life stage experiences, like marriage and pregnancy. Thus, the relationship between WASH practices and RTIs might vary across female reproductive life stages. This cross-sectional study assessed the relationship between WASH exposures and self-reported RTI symptoms in 3,952 girls and women from two rural districts in India, and tested whether social exposures represented by reproductive life stage was an effect modifier of associations. In fully adjusted models, RTI symptoms were less common in women using a latrine without water for defecation versus open defecation (Odds Ratio (OR) = 0.69; Confidence Interval (CI) = 0.48, 0.98) and those walking shorter distances to a bathing location (OR = 0.79, CI = 0.63, 0.99), but there was no association between using a latrine with a water source and RTIs versus open defecation (OR = 1.09; CI = 0.69, 1.72). Unexpectedly, RTI symptoms were more common for women bathing daily with soap (OR = 6.55, CI = 3.60, 11.94) and for women washing their hands after defecation with soap (OR = 10.27; CI = 5.53, 19.08) or ash/soil/mud (OR = 6.02; CI = 3.07, 11.77) versus water only or no hand washing. WASH practices of girls and women varied across reproductive life stages, but the associations between WASH practices and RTI symptoms were not moderated by or confounded by life stage status. This study provides new evidence that WASH access and practices are associated with self-reported reproductive tract infection symptoms in rural Indian girls and women from different reproductive life stages. However, the counterintuitive directions of effect for soap use highlights that causality and mechanisms of effect cannot be inferred from this study design. Future research is needed to understand whether improvements in water and sanitation access could improve the practice of safe hygiene behaviors and reduce the global burden of RTIs in women.

## Introduction

Girls and women experience greater challenges than boys and men in safely accessing water, sanitation, and hygiene (WASH) resources, including social and sexual violence, while seeking locations to address bodily needs.[1–7] In addition, women have greater needs for consistent access to sanitation and water to maintain personal hygiene, particularly during menstruation. Inadequate water and sanitation access affects women’s health in many ways beyond infectious disease, including increased psychosocial stress, urinary incontinence and constipation, maternal mortality, and preterm birth.[5, 8–11] Water and sanitation access may also be important determinants of hygiene-related diseases, like reproductive tract infections (RTI).

The worldwide burden of RTIs in women is high, affecting as many as a third of all women of reproductive age in some regions of the world.[12] RTIs are a group of etiologically distinct diseases that share a common set of non-specific symptoms caused by inflammation and host immune responses.[13, 14] The most common symptoms for vaginitis, a leading cause of RTIs worldwide, includes abnormal vaginal discharge, vulvar itching and irritation, and malodor, although asymptomatic disease is also very common.[15] Early prevention of RTIs is critical because they can increase the risk of other severe reproductive diseases, including pelvic inflammatory disease, infertility, sexually transmitted diseases, ectopic pregnancy, miscarriage, and preterm birth.[5, 8, 16–24] RTI symptoms can be caused by sexually transmitted infections, like trichomoniasis, as well as by bacterial vaginosis and vaginal candidiasis, which have been linked to both sexual and vaginal hygiene exposures.[13, 25, 26] Hygiene practices, including frequency of bathing, douching, using a cloth to clean inside the vagina, type of cleansing material, quality of bathing water, and washing and reusing cloth pads as an absorbent material during menstruation have been implicated as risk factors for self-reported and diagnostically-confirmed vaginitis.[27–35]

Inadequate access to a private sanitation location with water for vaginal and anal cleansing may make it more difficult for women to maintain both daily and menstruation-specific vaginal hygiene behaviors, which could lead to chronically unhygienic vaginal conditions.[3, 34] Few published studies have explored whether water and sanitation access, and related daily hygiene practices (not specific to menstruation or sexual activity) affects the risk of RTI disease. One case-control study linked to this study found that after accounting for the use of cloth pads and socio-economic factors, water and sanitation access was not associated with RTI symptoms or laboratory confirmed vaginosis in women presenting for care at a health care center.[32] Yet RTIs are a grossly unreported disease and socio-economic, education, and WASH risk factors may differ between women seeking care at a health care center versus the broader population, especially for rural women with the lowest levels of WASH worldwide.[36] Knowledge on risk factors among low-income, rural women and girls is limited, in part because they often are physically or economically disadvantaged in accessing health care centers with laboratory infrastructure and personnel for disease diagnosis. Two population-based studies in India reported household water and sanitation was associated with RTIs in unadjusted analysis, but neither study reported effects after adjusting for other potential confounders.[34, 37] If water and sanitation access is an important determinant of RTI risk in women, then global efforts to improve women’s water and latrine coverage may reduce the burden of RTIs among the most vulnerable women worldwide.

Since RTI symptoms can be caused by a variety of sexual and hygiene-related diseases, disentangling the impact of WASH versus social or sexual exposures on RTI risk can be challenging. In addition to the WASH risk factors above, marriage, frequent sexual contact, pregnancy, biological age, and use of intrauterine contraceptive devices (IUDs) for family planning are also risk factors for an RTI. [34, 35, 38–40] These socio-sexual risk factors are likely to be correlated with each other, and with WASH practices linked to specific reproductive life stages, such as menarche, marriage, and pregnancy. Transitions between life stages, from menarche to menopause, can increase or decrease a woman’s access to wealth, education, environmental resources (like WASH), and social interactions.[3] Thus, life stage could modify the risk of RTI disease across a woman’s reproductive life course. Examining the impact of WASH practices on RTI disease at different female life stages could improve understandings about the potential efficacy and targeting of interventions for RTI disease burden in women and girls. The objective of this cross-sectional study was to evaluate whether WASH practices were associated with self-reported RTI symptoms in girls and women in rural regions of India, and whether associations varied across stages of the socially-defined reproductive life stages.

## Methods

### Ethical considerations

Written informed consent was obtained from all participants prior to data collection. The study was approved by the scientific and ethical review committees at the Asian Institute of Public Health, Emory University, the London School of Hygiene and Tropical Medicine, and the University of Oklahoma.

### Study setting and design

We conducted a cross-sectional, population-based surveillance survey between September 2013 and March 2014 in Odisha, India, an area of India with particularly low levels of water and sanitation coverage, and high maternal and child morbidity and mortality (S1 Table, S1 Dataset).[41] The study was nested within a broader study entitled “Life course approach for exploring the impact of sanitation access and menstrual hygiene management (MHM) on psychosocial stress, behavior, and health among girls and women in Odisha (Orissa), India”.[3, 4, 10, 32] To increase variability in our population, the study was conducted in two non-connected rural districts of Odisha, which included 152 coastal villages in Khorda District and 157 inland villages in Sundargarh District.

### Sample size

Data were collected as part of a larger population-based survey used to identify and recruit women in the first trimester of pregnancy for a cohort study of sanitation access and adverse pregnancy outcomes.[10] In order to meet sample size requirements for the cohort study (N = 670), a total of 4,020 women were surveyed.

### Data collection and management

Inclusion criteria were being female, reporting experiencing menstrual periods, and being between the ages of fourteen and forty-five years, which falls between the mean age of menarche (13.6 years) and menopause (46.1 years) in Indian women.[42, 43] Women trained as Community Health Volunteers (CHVs) from study villages were engaged to identify households and one eligible participant was randomly selected from each household, without replacement, and asked for consent to participate in the study. CHVs administered a structured survey in the local language in a location that offered privacy to the subject and recorded responses on paper forms. Survey responses were entered by two data entry personnel using EpiInfo (Center for Disease Control, Atlanta, GA) and were cross-checked for consistency.

### Outcome

Our primary outcome of interest was symptoms of a RTI, assessed based on self-report of unusual vaginal discharge, itching, or irritation in the previous two weeks. Self-reported symptoms were ultimately used to determine outcome status because a) our primary study population was rural women with extremely low WASH access, most of whom lived far from health care centers with diagnostic laboratories; b) transportation and processing of thousands of swabs from this geographically dispersed set of villages was logistically and economically unfeasible; and c) most importantly, initial evaluation suggested we would experience challenges in recruiting asymptomatic women into a study involving collection of vaginal swabs during a household visit, which would result in skewed sampling of information across the population. To improve the quality of self-reported data, an easily recognized group of symptoms with modest sensitivity and specificity in predicting the presence of bacterial vaginosis and other RTI diseases like vulvovaginal candidiasis and trichomoniasis vaginalis was selected.[44–50] Recall of disease symptoms was limited to two weeks to reduce the potential for self-recall bias. If a woman reported “yes” to any of these symptoms, she was categorized as positive for an RTI.

### Socioeconomic confounders

*A priori* selected confounders included religion, level of educational attainment, caste, occupation, and ownership of a Below-Poverty-Line (BPL) card as a proxy for household wealth (Table 1).

**Table 1:** Definition of confounder and exposure variable levels.

Variable	Level	Definition
Socio-economic confounders		
Religion	Hindu	
	Muslim	
	Christian	
	Other	
Occupation	Employed or self-employed	
	Housewife	
	Student	
	Other	
Education	None	No formal education
	Primary	Completed Primary education
	Secondary	Completed Secondary education
Poverty	No BPL card	
	BPL card	
Exposures of Interest		
Drinking water source	Household Improved water	Piped tap, tube well, borehole, protected spring, rainwater, or protected dug well that is available on a daily basis and is located in house or yard
	Other Improved water	Piped tap, tube well, borehole, protected spring, rainwater, or protected dug well that is available on a daily basis and is located outside house or yard but within 30 minutes round trip travel time ^1^
	Unimproved	Any water type that requires more than 30 minutes round trip to collect, is not available daily, or is of unimproved type, including rivers, lakes, ponds, or unprotected wells or springs ^1^
Sanitation Access	Latrine with water	Defecates in private or shared latrine with water source
	Latrine without water	Defecates in private or shared latrine
	No latrine	Defecates in open areas
Distance to defecation location	< = 10 min.	Less than 10 minutes one way ^2^
	> 10 min.	Further than 10 minutes one way ^2^
Handwashing location	Household	On premise—In or near toilet facility/in or near kitchen/elsewhere
	Outside	Outside premises/no specific place
Handwashing on any occasion	Detergent, soap	Detergent or soap & water
	Other	Ash, Soil, or mud and water
	Water only or no wash	Do not wash hands or use water only
Handwashing after defecation	Detergent, soap	Detergent or soap & water
	Other	Ash, Soil, or mud and water
	Water only or no wash	Do not wash hands or use water only
Personal bathing frequency	Daily	At least once a day
	Not daily	Less than once a day
Bathing water source	Improved	Piped tap, tube well, borehole, protected spring, rainwater, or protected dug well that is available on a daily basis and is located outside house or yard but within 30 minutes round trip travel time
	Unimproved	Any water type that requires more than 30 minutes round trip to collect, is not available daily, or is of unimproved type, including rivers, lakes, ponds, or unprotected wells or springs
Distance to bathing location	< = 7 min.	Less than 7 minutes ^3^
	> 7 min.	Further than 7 minutes ^3^
Materials used for day to day cleansing	Soap	
	Water only	
Location used for menstrual hygiene management	Toilet	Toilet
	Room	Private room in house
	Open	Open area outside the household
Absorbent Materials	Disposable	Disposable sanitary pads/tampons
	Reusable	Reusable cloths/towels
Life stage Group	Unmarried youth	Single marital status and less than 24 years of age
	Newly Married	Married for 2 or less years
	Pregnant	Pregnant woman, regardless of age or marital status
	Established Married	Married for more than 2 years
	Other	Single/divorced/widowed/separated marital status and/or over 24 years of age

^1^ Cut point of 30 minutes used to define water source based upon WHO/UNICEF JMP definitions for improved water.

^2^ Cut point of 10 minutes selected based upon median reported time for women in this population to travel to defecation site.

^3^ Cut point of 7 minutes selected based upon median reported time for women in this population to travel to bathing source.

### Exposures

Our primary exposures of interest (Table 1) were the subject’s current (not restricted to past two weeks) WASH practices that could influence their ability to consistently maintain vaginal cleanliness and dryness. Variables included access to an "improved” drinking water source as defined WHO/UNICEF by the Joint Monitoring Programme for Drinking Water Supply and Sanitation (JMP) for post-2015 monitoring, primary use of a latrine for defecation, the number of minutes to travel to that defecation location one way, and consistency in use of a latrine over the last month among those that used a latrine.[51] Initial analysis of household latrine access discovered sparse numbers of households using a shared or other unimproved latrine, so a binary variable for any versus no latrine access was created. Hygiene behaviors included where the participant bathes, how often they bathe, the quality of water used for bathing (from an improved water source), distance to the bathing location, materials used for cleansing the body, general and post-defecation handwashing practices, and type of handwashing materials.[51] MHM variables included type of absorbent used during menstruation and having access to a private location to manage menstrual hygiene, based upon association between these factors and symptoms of a RTI or laboratory-confirmed urinary tract infection or bacterial vaginosis in non-pregnant women at a health care facility.[38] Samples of survey questions are provided in S2 Table.

A secondary exposure and effect modifier of interest included reproductive life stages that represent significant changes in a woman’s social and physical environment, sexual activity (marriage), or biological state (pregnancy) after menarche. Life stages were defined based on a woman’s age and marital and pregnancy status at the time of data collection.[4] *Unmarried youth* were unmarried women between 14 and 24 years of age who had reached menarche and lived with their parents or guardians. *Newly married women* were women who were married for less than two years and were living with their husband’s family. Sexual activity among unmarried women is rare, so shifts between adolescence to newly married status reflect the onset of sexual activity in a woman’s life.[52, 53] *Pregnant women* included women in all gestational weeks of fetal development, regardless of parity. *Established adult women* were married more than two years, regardless of age, and were not currently pregnant. *Other women* were over 24 years of age and divorced, separated, never married and not living at home, or widowed.

### Statistical analysis

Data were analyzed using SAS version 9.4 (SAS Institute, Carey, NC). Data analysis was limited to subjects for whom responses were available on symptoms of a RTI. Two variables were noted to have missing data. A confounding variable for caste of subjects was not included in imputation and analysis because more than 25% of the values were missing. Prior to conducting analyses, multiple imputation was employed to impute for availability of water in a latrine due to missing information for 4.6% of subjects with complete outcome data.[54, 55] Multiple imputation considers the distribution of the non-missing observations and draws a random sample from that distribution to impute the missing values. Ten independent data sets were created and each of these datasets were analyzed separately. To complete the analysis, the results from the 10 analyses were combined to obtain pooled estimates. This method of imputation results in inferences that appropriately account for the uncertainty associated with missing data. After imputing the missing values for these categorical variables, the between-imputation variance was assessed and confirmed to be zero. Therefore, we produced estimates based on analyzing a single imputed dataset rather than pooling estimates from the ten imputed datasets. Descriptive statistics were reported as percentages.

To quantify the associations between RTI symptoms, WASH exposures, and life stage group we used generalized mixed logistic regression models (SAS Version 9.4, proc glimmix) with binary log link and a random intercept term to account for variance between districts. Effect modification of life stage group on associations between exposures and RTI symptoms was tested by including interaction terms in bivariate models and assessing for statistically significant interaction (*P*<0.05). No interaction term with life stage was significant, so associations between risk factors and RTIs are presented for all life stage groups combined. Multivariable model selection technique involved including all socio-economic confounder (SES) and exposure variables (Table 1) into a fully adjusted model and conducting backwards selection. Confounder variables for district, religion, education, occupation, and poverty were retained in all models during model selection. At each step of the model process, the exposure variable with the largest *p*-value for the overall effect of the variable on the outcome was removed and the beta coefficients for exposures and Akaike information criterion (AIC) values was used to assess model fit compared to previous models. Backwards selection was repeated until only district, SES confounders, and the WASH or life stage exposure variables that were associated with the lowest model AIC score remained. As a final model fitting step, interaction terms between WASH exposures were considered. Collinearity was assessed by computation of condition index diagnostics and variance decomposition proportions (VDPs), using condition indices >10 and VDPs >0.5 as an indication of collinearity. To reduce the risk of type I error from multiple comparison tests, a Bonferroni correction was used to estimate conservative CIs.

## Results

### Socio-economic and WASH exposures by life stage group

Systematic sampling identified 1,180 unmarried youth, 76 newly married, 371 pregnant, 2,148 established married, and 196 other (widowed, divorced, and never married) women. Complete data on exposures and health outcomes was analyzed for 3,952 women (missing for 19 (<0.5%)) between 14 and 45 years age from rural Khorda District (N = 2,824) and Sundargarh District (N = 1,147). Differences in socioeconomic confounders are shown by life stage group in Table 2.

**Table 2:** Site-stratified frequencies for socioeconomic confounders by life stage group.

Site	Level	Life Stage Group				
Exposure		Unmarried youth	Newly Married	Pregnant	Est. Married	Other
Sample size		N = 1,171	N = 75	N = 371	N = 2,139	N = 196
Religion ^1^						
	Hindu, n = 2,792	889 (75.9%)	52 (69.3%)	278 (74.9%)	1,443 (67.5%)	130 (66.3%)
	Muslim, n = 211	75 (6.4%)	2 (2.7%)	9 (4.6%)	103 (4.8%)	22 (5.9%)
	Christian, n = 935	203 (17.3%)	21 (28.0%)	68 (18.3%)	587 (27.4%)	56 (28.6%)
Occupation ^2^						
	Employed or self-employed, n = 449	170 (14.5%)	2 (2.7%)	20 (5.4%)	180 (8.4%)	77 (39.3%)
	Housewife, n = 2,363	0	71 (94.7%)	335 (90.3%)	1,935 (90.5%)	22 (11.2%)
	Student, n = 600	579 (49.4%)	0	9 (2.4%)	0	12 (6.1%)
Education						
	None, n = 724	55 (4.7%)	15 (20.0%)	60 (16.2%)	561 (26.2%)	33 (16.8%)
	Primary, n = 764	89 (7.6%)	11 (14.7%)	117 (31.5%)	522 (24.4%)	25 (12.8%)
	Secondary, n = 2,464	1,027 (87.7%)	49 (65.3%)	194 (52.3%)	1,056 (49.4%)	138 (70.4%)
Poverty	BPL card, n = 2,081	733 (62.6%)	37 (49.3%)	194 (52.3%)	1,017 (47.6%)	100 (51.0%)

Established (Est.); Minutes (min.).

^1^ “Other” of n = 14 not shown.

^2^ “Other” of N = 540 not shown.

Many WASH practices varied across the life stages of girls and women in this study (Table 3). Use of improved water sources for drinking was highest among pregnant and other types of women (Table 3). Primary use of latrines for defecation was higher among newly married, pregnant, and other women. More than half of all women reported walking more than ten minutes one way to their defecation site, but distance did not vary by group. Half of girls and women used water only to wash hands for most occasions, although newly married and other women were more likely to use soap or detergent. Use of soap, detergent, or ash/soil/mud was much more common for washing hands after defecation, in particular soap or detergent among pregnant women and ash/soil/mud among established married women. Nearly all pregnant women reported bathing daily, compared to about two-thirds of women from other life stage groups, most of whom reported using soap for bathing. Pregnant and “Other” women were most likely to use water from an improved water source to bathe and to report walking < 7 minutes to their bathing location. Pregnant were more likely to use soap or detergent for bathing. Among non-pregnant women, most reported using a private location in the household for menstrual hygiene management (including changing pads and washing pad materials), with newly married being most likely to use an open location outside the household. Unmarried youth and other women groups were more likely to use disposable pads or tampons than established married women who reused cloth pads.

**Table 3:** Chi squared P value for trend in differences in frequencies of water, sanitation, and hygiene practices by life stage group.

Site	Level	Life Stage Group					
Exposure		Unmarried youth	Newly Married	Pregnant	Est. Married	Other	P Value
Sample size		N = 1,171	N = 75	N = 371	N = 2,139	N = 196	
Drinking water access							<0.0001
	Household Improved water, n = 1,629	460 (39.3%)	28 (37.3%)	167 (45.0%)	880 (41.1%)	94 (48.0%)	
	Other Improved water, n = 1,989	641 (54.7%)	41 (54.7%)	165 (44.5%)	1,047 (49.0%)	95 (48.5%)	
	Unimproved, n = 334	70 (6.0%)	6 (8.0%)	39 (0.5%)	212 (9.9%)	7 (3.6%)	
Sanitation Access							0.0003
	Latrine with water supply, N = 210	53 (4.5%)	6 (8.0%)	35 (9.4%)	100 (4.7%)	16 (8.2%)	
	Latrine without water, N = 548	171 (14.5%)	11 (14.7%)	64 (17.3%)	271 (12.7%)	31 (15.8%)	
	No latrine, N = 3,209	947 (80.9%)	58 (77.3%)	272 (73.3%)	1,768 (82.7%)	149 (76.0%)	
Distance to defecation location	< = 10 min., n = 2,064	618 (52.8%)	41 (54.7%)	190 (51.2%)	1,109 (51.9%)	106 (54.1%)	0.9292
Handwashing location	In household, n = 1,229	372 (31.8%)	25 (33.3%)	130 (35.0%)	644 (30.1%)	58 (29.6%)	0.3672
Handwashing at any time							0.5835
	Detergent, soap, n = 1,963	582 (49.7%)	43 (57.3%)	183 (49.3%)	1,047 (49.0%)	108 (55.1%)	
	Ash, Soil, Mud, n = 40	10 (0.9%)	0 (0%)	5 (1.4%)	22 (1.0%)	3 (1.5%)	
	Water only or no wash, n = 1,949	579 (49.4%)	32 (42.7%)	183 (49.3%)	1,070 (50.0%)	85 (43.4%)	
Handwashing after defecation							<0.0001
	Detergent, soap, n = 2,424	754 (64.4%)	47 (62.7%)	290 (78.2%)	1,203 (56.2%)	130 (66.3%)	
	Ash, Soil, Mud, n = 710	197 (16.8%)	13 (17.3%)	39 (10.5%)	431 (20.2%)	30 (15.3%)	
	Water only or no wash, n = 818	220 (18.8%)	34 (17.4%)	42 (11.3%)	505 (23.6%)	36 (18.4%)	
Bathing frequency	Daily, n = 2,707	776 (66.3%)	127 (64.8%)	370 (99.7%)	1,386 (64.8%)	127 (64.8%)	<0.0001
Bathing water source	Improved Source, n = 2,528	760 (64.9%)	49 (65.3%)	261 (70.4%)	1,325 (61.9%)	133 (67.9%)	0.0163
Distance to bathing location	< = 7 min., n = 1,928	624 (53.3%)	37 (49.3%)	196 (52.8%)	1,050 (49.1%)	117 (59.7%)	0.0172
Material used for day to day cleansing							0.0025
	Soap, n = 3,364	993 (84.8%)	63 (84.0%)	342 (92.2%)	1,802 (84.2%)	164 (83.7%)	
	Other, n = 28	8 (0.7%)	0	12 (3.2%)	5 (0.2%)	3 (1.5%)	
	Water only, n = 560	170 (14.5%)	29 (14.8%)	17 (4.6%)	332 (15.5%)	29 (14.8%)	
Location for MHM							0.1777
	Latrine, n = 483	178 (15.1%)	13 (17.1%)	NA	262 (12.2%)	30 (15.3%)	
	Private location in home, n = 2,876	921 (78.1%)	56 (73.7%)	NA	1,743 (81.2%)	156 (79.6%)	
	Open site, n = 241	81 (6.9%)	7 (9.2%)	NA	143 (6.7%)	10 (5.1%)	
Absorbent Material	Disposable, n = 1,325	683 (57.9%)	31 (40.8%)	NA	511 (23.8%)	100 (51.0%)	<0.0001

P value is Chi Squared test for trend. Established (Est.); Minutes (min.).

### Risk factors for RTI symptoms in girls and women

Self-reported symptoms of abnormal vaginal discharge, itching, and irritation were reported by 402 (10.2%) of girls and women overall. Prevalence was lowest in unmarried youth (n = 96, 8.1%) and other women (n = 16, 8.2%), followed by established married (n = 224, 10.5%), newly married (n = 10, 13.3%), and pregnant (n = 57, 15.4%) women. Many WASH exposures were associated with RTI symptoms in bivariate analysis (Table 4). Although frequencies of many WASH exposures varied between life stage groups (Table 3), a woman’s life stage status did not modify the association between WASH exposures and RTI symptoms (Table 5). In a fully adjusted model including all confounders and exposures, many variables were not associated with RTI symptoms (Table 4). The best fitting model of RTI symptoms, adjusted for district and SES confounders, included variables for sanitation access, type of material used for hand washing after defecation, distance to bathing location, daily bathing, and bathing material, plus interaction terms for bathing material with post-defecation handwashing material (p = 0.002) and bathing material with poverty status (p = 0.003). Interaction terms for Life Stage Status and WASH conditions did not improve model fit and were not retained in the fparsimonious model. RTI symptoms were less common in women using a latrine for defecation versus open defecation (final Odds Ratio (fOR) = 0.69; 95% Confidence Interval (CI) = 0.58, 0.99), although there was no association with using a latrine with a water source (fOR = 1.09; CI = 0.69, 1.72). Symptoms were also less likely for women who walked seven minutes or less to their bathing location versus more than seven minutes (fOR) = 0.79; CI = 0.63, 0.99). Post-defecation handwashing material was an effect modifier for the relationship between bathing maternal and symptoms of RTI (p = 0.0034). Symptoms were less common among those who reported bathing with soap versus water among women who reported washing hands with soap after defecation (fOR = 0.81; 95% CI = 0.54, 1.24). However, symptoms were more common among those who bathed with soap if hands were washed with ash or mud (fOR = 1.56; 95% CI = 0.78, 3.13) or water only (fOR = 6.30; 95% CI = 1.94, 20.43) after defecation.

**Table 4:** Associations between water, sanitation, and hygiene variables, social life stage status, and reported symptoms of abnormal vaginal discharge, itching, and irritation in 3,952 girls and women in Odisha, India.

Exposure	Categorical Level	n/N (%)	Bivariate Model OR (95% CI)	Fully Adjusted Model OR (95% CI) ^1^	Final Model OR (95% CI) ^1^
**Drinking water access**					
	Household Improved water	162/1,629 (9.9%)	0.89 (0.71, 1.11)	0.85 (0.54, 1.35)	
	Other Improved water	207/1,989 (10.4%)	1.10 (0.89, 1.36)	0.87 (0.58, 1.32)	
	Unimproved	33/334 (9.9%)	Ref.	Ref.	
**Sanitation Access**					
	Latrine with water supply	26/210 (12.4%)	1.16 (0.75, 1.78)	1.13 (0.69, 1.83)	1.07 (0.68, 1.69)
	Latrine without water	48/548 (8.8%)	0.79 (0.58, 1.09)	0.72 (0.49, 1.05)	0.69 (0.49, 0.98)
	No latrine	328/3,194 (10.3%)	Ref.	Ref.	Ref.
**Distance to defecation location**					
	< = 10 min.	196/2,064 (9.5%)	0.85 (0.69, 1.04)	0.91 (0.71, 1.16)	
	> 10 min.	206/1,888 (10.9%)	Ref.	Ref.	
**Handwashing location**					
	Household	127/1,229 (10.3%)	0.94 (0.74, 1.19)	0.85 (0.66, 1.09)	
	Outside	275/2,723 (10.1%)	Ref.	Ref.	
**Handwashing at any time** ^**2**^					
	Soap or ash	208/2,003 (10.4%)	1.09 (0.89, 1.35)	1.06 (0.84, 1.33)	
	Water only or no wash	194/1,949 (10.0%)	Ref.	Ref.	
**Handwashing after defecation**					
	Soap	259/2,424 (10.7%)	1.53 (1.14, 2.06)	1.53 (1.12, 2.11)	^**3**^
	Other	85/710 (12.0%)	1.71 (1.20, 2.43)	1.72 (1.20, 2.46)	^**3**^
	Water only or no wash	58/818 (7.1%)	Ref.	Ref.	^**3**^
**Bathing frequency**					
	Daily	293/2,707 (10.8%)	1.27 (1.01, 1.60)	1.20 (0.94, 1.52)	
	Not daily	109/1,245 (8.8%)	Ref.	Ref.	
**Bathing water source**					
	Improved	271/2,528 (10.7%)	1.12 (0.89, 1.41)	1.23 (0.95, 1.61)	
	Unimproved	131/1,424 (9.2%)	Ref.	Ref.	
**Distance to bathing location**					
	< = 7 min.	194/1,928 (9.6%)	0.80 (0.64, 0.99)	0.79 (0.61, 1.02)	0.79 (0.63, 0.99)
	> 7 min.	208/2,024 (10.8%)	Ref.	Ref.	Ref.
**Material used for regular bodily washing** ^**2**^					
	Soap or Other	360/3,364 (10.7%)	1.52 (1.09, 2.13)	1.33 (0.94, 1.87)	^**3**^
	If washes hands after defecation with soap				0.81 (0.54, 1.24)
	If washes hands after defecation with ash or mud				1.56 (0.78, 3.13)
	If washes hands after defecation with water				6.30 (1.94, 20.43)
	Water only	42/588 (7.1%)	Ref.	Ref.	Ref.
**Location for MHM**					
	Toilet	62/601 (10.3%)	1.08 (0.62, 1.85)	NC	
	Private	316/3,072 (10.3%)	1.09 (0.68, 1.73)	NC	
	Open site	24/279 (8.6%)	Ref.	NC	
**Absorbent Pad**					
	Disposable	141/1,514 (9.3%)	0.79 (0.62, 1.00)	NC	
	Reusable	261/2,438 (10.7%)	Ref.	NC	
**Life stage Group**					
	Unmarried youth	95/1,171 (8.1%)	Ref.	Ref.	
	Newly Married	10/75 (13.3%)	1.78 (0.88, 3.57)	1.27 (0.53, 3.07)	
	Pregnant	57/371 (15.4%)	2.02 (1.42, 2.87)	1.26 (0.67, 2.38)	
	Established Married	224/2,139 (10.5%)	1.34 (1.04, 1.73)	0.95 (0.53, 1.70)	
	Other	16/196 (8.2%)	1.02 (0.59, 1.77)	1.01 (0.56, 1.83)	
	AIC (DF)			2562.700 (27)	2530.980 (19)

Odd ratios (OR) and Bonferroni-corrected 95% confidence intervals (CI). MHM: Menstrual Hygiene Management NC: Not calculated due to absence of data for pregnant women; Ref.: Reference group; Akaike information criterion (AIC).

^**1**^ Odds ratios adjusted for district, religion, education, occupation, and poverty status.

^**2**^ Categories for washing with “other” materials were combined with soap due to sparse number of responses.

^**3**^ Final model includes interaction term for bathing material with post-defecation handwashing material, and effects for bathing material are presented by category of the post-defecation handwashing material effect modifier.

**Table 5:** Assessment of interaction between life stage group and water, sanitation, and hygiene exposures on symptoms of RTIs.

Water, sanitation, and hygiene covariate	Degrees of Freedom for interaction term	Wald Chi Square	P Value for Type 3 Analysis of Effects
Improved drinking water source	8	4.2557	0.8333
Defecation Location	8	5.6588	0.6793
Distance to defecation location	4	7.7116	0.1027
Handwashing location	4	4.3276	0.3635
Handwashing on any occasion	7	3.1996	0.8659
Handwashing after defecation	8	10.6754	0.2208
Personal bathing frequency	4	3.5086	0.4766
Bathing water source	4	1.4634	0.8331
Distance to bathing location	4	5.4407	0.2450
Materials used for day to day cleansing	4	4.1180	0.3903
Location used for MHM (excluding pregnant women)	8	6.0387	0.8747
Absorbent Materials (excluding pregnant women)	4	2.5879	0.5644

## Discussion

This study sought to understand the relationships between WASH practices and two-week prevalence of RTI symptoms across reproductive life stages of girls and women in Odisha, India. We demonstrated that self-reported symptoms of RTI disease were less common in girls and women with access to a latrine (vs open defecation) and lower walking times to a bathing location (< 7 minutes vs > 7 minutes). The lower prevalence of RTIs among latrine users may reflect reduced exposure to infectious vaginosis (e.g. *Gardnerella vaginalis*) or vaginal candidiasis microbes in soil or water at open defecation areas.[13, 25, 26] Women in this rural population perceive open sites to be causes of RTI symptoms.[4] Detection of *G*. *vaginalis* in soil or water to vagina has never been described, although transmission of *Candida spp*. by soil or water is possible.[56] Rather than environmental transmission of invasive microbes, lack of access to a latrine and nearby water supply might promote unhygienic defecation, urination, and bathing practices that lead to genital uncleanliness, which can promote pathogen infection or a polymicrobial imbalance of vaginal microbiota. The journey to find a safe, private location for defecation and urination is often stressful and physically challenging for women, and can require walking long distances through unsafe terrain while carrying water for cleansing.[3, 38] Women may attempt to reduce this stress by carrying less water for genital washing or bathing less frequently, which has been a risk factor for RTIs in other studies [27, 29, 35, 40, 57]. Similarly, women forced to spend more time to reach a location with water for bathing may decrease the frequency or quality of time spent on personal hygiene.[58] Menstruation poses an additional set of social and physical restrictions that limit the frequency of bathing, like restricted access to a water supply, lack of private space for MHM, and health beliefs that frequent bathing might cause problems in future pregnancies.[59] Having a private space for MHM was associated with a lower likelihood of laboratory-confirmed bacterial vaginosis in our related case-control study.[32] MHM factors could not be included in adjusted models in this study due to the inclusion of pregnant subjects, but MHM practices may have contributed to RTIs in non-pregnant subjects. Based upon the fact that pregnant women were the most likely to report symptoms, MHM practices are unlikely to be the only trigger of acute RTI symptoms.

Elevated risk for RTI symptoms in pregnant women is common due to changes in placental microbial composition and immune responses, which highlights the issue that immunological competence plays a key role in susceptibility, as well as symptomology of RTI disease.[14, 60] Reported symptoms of an RTI may actually be more of an indicator of susceptibility to vaginitis from immune dysregulation or suppression. In the context of our study, that would mean that women practicing open defecation or using distant bathing locations are less capable of resisting infection or maintaining vaginal homeostasis than women with latrines or nearby bathing locations. Women who defecate or bathe in public areas may be more likely to be infected by helminth or diarrhea pathogens that can suppress general mucosal responses, including those that regulate vaginal microbiota homeostasis and promote immune clearance of pathogens. Another possibility is that women who must leave the home and address hygiene needs in public locations are more likely to experience biological effects from chronic or elevated psychosocial stress.[3] Stress can cause immune suppression and dysregulation that disrupts the body’s ability to regulate vaginal homeostasis or resist RTI infections.[61] Chronic and early life psychosocial stressors, including discrimination and poverty, have been linked to bacterial vaginosis in pregnant women in the United States and to symptoms of RTIs in women in India.[62–65] Gynecological disorders have been also been linked to mixed anxiety-depressive disorder in married Indian women, mental distress in married Lebanese women, occupational stress among Chinese factory workers, and post-war depression and post-traumatic stress disorder in US veterans.[66–69] Alleviation of chronic WASH-related stress may be important for reducing the risk of RTIs in women.

Related to these disease pathways, we had hypothesized that WASH practices and the related risk of RTIs would change for women as they transition through life stages representing different social and sexual roles, from unmarried youth to marriage and pregnancy and finally matriarchy. To our knowledge this is the first study to structure analysis of risk factors for RTIs based upon *a priori* hypotheses that environmental exposures for women in settings like India can be moderated by social life stages. Although WASH practices did vary for women from different life stage groups, no evidence was found that life stage modified or confounded the association between RTI symptoms and WASH exposures. Furthermore, life stage was not associated with RTIs after adjusting for SES and WASH factors–a surprising finding given reports from other studies that factors related to sexual activity and reproduction, such marriage, pregnancy, biological age, and use of intrauterine contraceptive devices (IUDs), can elevate RTI risk.[32, 34, 35, 39, 40] Our study instead found that associations between WASH conditions and RTI symptoms were static across reproductive life stages representing menarche to menopause. This points to the need for interventions to address WASH access for women throughout all stages of the reproductive life cycle.

The associations between RTIs and washing hands after defecation or bathing with soap is less clear. Post-defecation hand washing has not been assessed in prior RTI studies, and there isn’t a clear biological mechanism for this relationship. In this study, post-defecation hand washing practices were an effect modifier of the relationship between type of material used for bathing of the body and symptoms of an RTI, with use of soap for bathing trending towards protective among post-defecation soap hand washers versus risky for post-defecation ash, mud, or water only hand washers. Some studies have reported that infrequent use of soap for vaginal bathing is a risk factor for RTIs, while others reported that frequent use of soap for vaginal washing, especially inside the vagina, increases the risk of RTIs via disturbance of the healthy vaginal microbiota.[12, 70–72] Soap use for post-defecation hand washing or for bodily bathing, both desirable, promoted hygiene practices, may have been over-reported among women with RTI symptoms.[73, 74] Alternatively, a proportion of women who had symptoms prior to the survey may have reacted to symptoms by changing their hand or body washing practices to mitigate feelings of disgust or shame, or to promote resolution of symptoms.[11, 75] A third possibility is that women who wash their hands or bodies with soap are more knowledgeable about health and health prevention and thus are more capable of accurate reporting of abnormal health symptoms. We adjusted for confounding from education or wealth on reporting of symptoms, although the indicators used may not be related to health and hygiene awareness knowledge and practices. For example, knowledge of healthy versus unhealthy reproductive conditions may be acquired more through social relationships with other women or health providers, rather than through traditional educational systems. Biased reporting or reverse causation might also be responsible for the effects observed for latrine access and bathing water distance, although the motivations for women with RTIs to *under*-report latrine use or bathing location, or react to symptoms by reverting from latrine to open defecation or moving farther away to bathe are less clear. As with soap ownership, women with latrines could be more knowledgeable about health and health prevention and be more likely to report abnormal symptoms, which in this case would strengthen our confidence that these women lack symptoms of an RTI.

While a cross-sectional study was a rapid and efficient way for exploring our hypotheses, this design cannot establish causal relationships between exposures and outcomes in this study. In addition to the above, other limitations include understanding whether exposures occurred early in childhood, prior to menarche, rather than in the weeks preceding this survey.[62] Retrospective questions about early life WASH exposures were considered, but recall of hygiene practices in early childhood was thought to be unreliable. This also includes the possibility that our questions about primary WASH access and practices were not the same practices used by the subject in the past two weeks–the window of time used to measure symptom prevalence. Furthermore, there isn’t a clear explanation for associations between WASH conditions and STIs, unless associations were proxies for differences in sexual practices between women with and without latrines and nearby bathing water sources. Adjusting for life stage status did not affect the WASH and RTI symptom relationship, suggesting the etiology of symptoms associated with WASH factors in this study are not sexual in origin.

Another major limitation of this manuscript was the use of self-reported symptoms as an outcome. The prevalence of reported symptoms in this study was low compared to similar population-based studies in Indian women (16% to 55%), although was higher than the 7.1% of lab-confirmed BV cases reported by a mobile clinic based study of rural women.[35, 37, 76, 77] Due to the prevalence of asymptomatic RTI disease, self-reported symptoms could have resulted in underestimation of total disease prevalence and nondifferential misclassification of some “diseased” women as “healthy”. These types of symptoms also could have been caused by STIs and resulted in overestimation of RTI prevalence and nondifferential misclassification of “healthy/RTI-negative” women as “diseased/RTI-positive”. Sexually transmitted diseases are considered rare in rural Indian women, so this latter scenario is unlikely.[77, 78] In both cases of health misclassification, similar rates of misclassification among exposed and unexposed would either result in unbiased estimates or bias of estimates towards the null.[79]

Clinic-based studies can optimize recruitment of symptomatic women and provide the infrastructure and personnel capable of performing laboratory assays for diagnostic confirmation of disease. However, clinic-based designs introduce significant recruitment bias that could limit generalizability of observations beyond certain populations of women. Seeking treatment at a health care center requires women to be self-aware of symptoms, and to be willing or able to seek treatment. Health care utilization for treatment of RTI symptoms among Indian women is often low (16% to 55%) due to lack of awareness of disease state, perception that symptoms are normal, fear of shame and embarrassment associated with symptomatic status, or restrictions on their ability to travel unaccompanied.[35, 76] Like other population-based studies, reported health care seeking behavior for RTI symptoms was low (13%) among the rural women in this population-based study. Laboratory diagnostics for improved outcome classification were deemed unfeasible for several reasons. Preliminary consideration suggested that proportional sampling of diseased and non-diseased women might be skewed due to resistance among presumptively healthy women to consent to invasive vaginal exams for this socially stigmatized disease.[76] Additionally, implementing diagnostic assays across such a broad geographic area of rural villages was cost and logistically prohibitive. Use of self-reported outcomes was deemed an acceptable limitation to ensure that we could systematically obtain data from populations of rural, low-income women with the poorest levels of WASH and health care access. This population-based (this study) was purposefully conducted in parallel with the Das et al. 2015 clinic-based study to ensure that our conclusions about risk factors for RTIs were drawn from a variety of populations and collectively accounted for various study design limitations.[38] As expected, levels of income, education, religion, health care utilization, and access to household water sources and latrines were much higher among women who sought treatment at health care centers in Das et al. than women from the same population recruited for this study. This highlights the importance of using mixed population and health care-based study designs for researching the determinants and burden of reproductive tract diseases in women in India.

Much of the focus in WASH interventions has historically centered on evaluating their impact on infectious disease in children. But this paper highlights that gender-specific outcome measures, like RTIs, might also be benefits of improvements in water and latrine access. Future research should explore the generalizability of these findings in other contexts and seek to understand the causal relationship between sanitation infrastructure, hygiene practices, and women’s health. Trials of water and sanitation interventions could collect information on indicators of women’s sanitation and hygiene practices and reproductive health to evaluate whether improvements in WASH reduce the burden of RTI disease in women. Reductions in RTI disease could have far reaching implications for other reproductive diseases, including pelvic inflammatory disease, infertility, sexually transmitted diseases ectopic pregnancy, miscarriage, preterm birth, and delivery of a low birth weight infant during pregnancy. [5, 8, 10, 16–24, 80] Accurate diagnosis of RTI disease remains a fundamental challenge to inclusion of reproductive health indicators in monitoring surveys. Longitudinal community-based studies employing molecular genomics approaches to characterize vaginal microbiota patterns linked to disease would help identify simple RTI indicators for surveillance needs and improve understandings about the relationship between WASH access and RTIs in women.

## Supporting information

S1 Table STROBE statement—Checklist of items that should be included in reports of observational studies. (DOC)

S2 Table SURVEY QUESTIONS. Description: These are the survey questions used to collect information about socio-economic confounders, WASH practices, 2 week self-reported symptoms of a reproductive tract infection, and life course status of participants.
(DOCX)

S1 Dataset De-identified subject data on outcome, exposures, and confounders used in this analysis. (XLSX)
