# Supplementary material for: From menarche to menopause: A population-based assessment of water, sanitation, and hygiene risk factors for reproductive tract infection symptoms over life stages in rural girls and women in India
Source: PLoS One. 2017 Dec 5;12(12):e0188234. doi: 10.1371/journal.pone.0188234 (PMC5716553; doi:10.1371/journal.pone.0188234)
Supplement: S2 Table — Description: These are the survey questions used to collect information about socio-economic confounders, WASH practices, 2 week self-reported symptoms of a reproductive tract infection, and life course status of participants. (DOCX) [file pone.0188234.s002.docx]

S2 Table. SURVEY QUESTIONS

Description: These are the survey questions used to collect information about socio-economic confounders, WASH practices, 2 week self-reported symptoms of a reproductive tract infection, and life course status of participants.

1. How old are you?
2. What is your marital status?
   1. Single, never married
   2. Married
      1. How many years have you been married?
   3. Widowed
   4. Divorced
   5. Separated
3. Are you currently pregnant?
   1. How many months are you pregnant?
4. What is your religion?
   1. Hindu
   2. Muslim
   3. Christian
   4. Other
5. What is the highest standard year of education that you have completed?
   1. No formal education
   2. Some primary (1-4th year)
   3. Completed primary (5th year)
   4. Some secondary (6-10th year)
   5. Completed +2 year (12th year)
   6. Completed +3 year (university, etc)
6. What is your occupation?
   1. Employed or self-employed
   2. Housewife
   3. Student
   4. Other
7. Does your household own a BPL card? (enumerator please verify)
   1. No
   2. Yes, verified
   3. Yes, not verified
8. What is the primary source of drinking water that you have used in the past month? (Select one)
   1. Piped water
   2. Hand pump
   3. Protected dug well
   4. Unprotected dug well
   5. Protect spring
   6. Unprotected spring
   7. Rainwater
   8. Tanker truck
   9. Surface water (River, dam, lake, Pond, stream, Canal, irrigation channel)
   10. Bottled water
   11. Other (specify):
9. Where is the water source located?
   1. In own dwelling
   2. In own compound
   3. In relative’s dwelling or compound
   4. In neighbor’s dwelling or compound
   5. In public place Outside compound
10. Is this water source available every single day?
    1. Yes
    2. No
11. What is the main source of water that you are using for body washing in the last month?
    1. Piped water
    2. Hand pump
    3. Protected dug well
    4. Unprotected dug well
    5. Protect spring
    6. Unprotected spring
    7. Rainwater
    8. Tanker truck
    9. Surface water (River, dam, lake, Pond, stream, Canal, irrigation channel)
    10. Bottled water
    11. Other (specify):
12. How many minutes does it take to go to this bathing water source?
13. How many minutes does it take you to go fetch water from this source and come back?
14. How often do you wash yourself (bath or vaginal wash)?
    1. Once a day
    2. Twice a day
    3. Other:
15. What do you use to wash yourself (bath or vaginal wash) during a normal day?
    1. Water only
    2. Water and soap or detergent
    3. Water and ashes
    4. Water and soil/dirt/mud
16. Where you normally wash your hands? (CHECK ALL THAT APPLY)
    1. In or near toilet facility
    2. In or near kitchen
    3. Elsewhere on premises
    4. Outside premises
    5. No specific place
17. What do you use most often to wash your hands? Are there any other materials you use?
    1. Do not wash hands
    2. Only water
    3. Detergent or Soap & water
    4. Ash and water
    5. Soil/dirt/mud and water
    6. Other (Specify):
18. What have you been using in the last month to wash your hands after defecation?
    1. Do not wash hands
    2. Only water
    3. Detergent or Soap and water
    4. Ash and water
    5. Soil/dirt/mud and water
    6. Other (Specify):
19. Where is the facility that you have been using for defecation in the last month? (Select one primary location)
    1. Facility in house or yard
    2. Facility in relative or neighbor’s house or yard
    3. Facility in community
    4. No facility–go in household and dispose of in (bush/field/ground/river/stream)
    5. No facility – go to bush/field/ground/river/stream
20. Are there any other locations? (Select all other locations)
    1. Facility in house or yard
    2. Facility in relative or neighbor’s house or yard
    3. Facility in community
    4. No facility–go in household and dispose of in (bush/field/ground/river/stream)
    5. No facility – go to bush/field/ground/river/stream
21. If using a facility, then hat kind of facility is the primary location? (CHECK ONE)
    1. Flush/pour flush to:
       1. piped sewer system
       2. septic tank
       3. pit latrine
       4. elsewhere
       5. unknown place/not sure/DK where
    2. Ventilated improved pit latrine (VIP or KVIP)
    3. Simple pit latrine with floor/slab
    4. Pit latrine without floor/slab or open pit
    5. Composting/dry latrine
    6. Service or bucket latrine (where excreta are manually removed)
    7. Hanging latrine
22. How many households, other than your own, are currently sharing this facility? Include households of neighbors and relatives. (Respond with a number, code 00 for “none”)
23. How many minutes does it take for you to walk to your primary defecation site? (If in household, then skip to next question).
24. Where is water collected that is used for flushing the toilet?
    1. From source inside facility
    2. Brought from private source outside
25. Where do you change your absorbent menstrual material? (select the main one)
    1. In a household toilet
    2. In a private room in the house
    3. In a neighbors/relatives/public facility outside the house or yard
    4. In the bush/field/ground/river/stream site
    5. Other
26. What absorbent material did you use most often during the last 6 cycles? (select the main one)
    1. disposable sanitary pads
    2. reusable cloths/towel
    3. tampon
    4. nothing
    5. other:
27. In the last two weeks have you experienced unusual vaginal itching, irritation, or discharge?
    1. Yes
    2. No
